# Ion channels and transporters in the development of drug resistance in cancer cells

**DOI:** 10.1098/rstb.2013.0109

**Published:** 2014-03-19

**Authors:** Else K. Hoffmann, Ian H. Lambert

**Affiliations:** Department of Biology, University of Copenhagen, 13 Universitetsparken, Copenhagen Ø 2100, Denmark

**Keywords:** cancer, drug resistance, tumour proliferation, apoptosis, ion channels in cancer

## Abstract

Multi-drug resistance (MDR) to chemotherapy is the major challenge in the treatment of cancer. MDR can develop by numerous mechanisms including decreased drug uptake, increased drug efflux and the failure to undergo drug-induced apoptosis. Evasion of drug-induced apoptosis through modulation of ion transporters is the main focus of this paper and we demonstrate how pro-apoptotic ion channels are downregulated, while anti-apoptotic ion transporters are upregulated in MDR. We also discuss whether upregulation of ion transport proteins that are important for proliferation contribute to MDR. Finally, we discuss the possibility that the development of MDR involves sequential and localized upregulation of ion channels involved in proliferation and migration and a concomitant global and persistent downregulation of ion channels involved in apoptosis.

## 1. Introduction

Multi-drug resistance (MDR) to chemotherapy is a major challenge in the treatment of cancer and is one cause of cancer chemotherapy failure. The cellular resistance of tumour cells to chemotherapeutic agents can be an innate property (termed intrinsic resistance) or can be acquired during chemotherapy (termed extrinsic resistance). Intrinsic resistance is often associated with cell differentiation or with genetic changes that occur during the initiation of tumour formation. Extrinsic resistance arises through the expansion of rare genetic variants in a tumour cell population owing to the proliferation of drug-resistant cells with selective advantages. As suggested by the name, MDR refers to resistance to numerous drugs that have different chemical structures and distinct mechanisms of action. Several molecular mechanisms have been proposed to explain MDR, including tumour cell-specific mechanisms such as decreased drug accumulation in the cell, sequestration of the drug in intracellular vesicles, activation of DNA repair pathways that counteract the effects of the drugs and evasion of apoptosis or cell cycle arrest [1–3]. Extracellular mechanisms have also been proposed, such as involvement of the stromal cell compartment in drug uptake and activation of alternative escape pathways. In addition, genes that control cell death and survival signalling, including the genes encoding Bcl-2 and p53, can acquire mutations that lead to drug resistance through modulation or impairment of apoptosis. Moreover, activation of alternative signalling pathways that modulate cell migration, proliferation and apoptosis may be involved in development of drug-resistance pathways [4,5].

**Figure 1.**
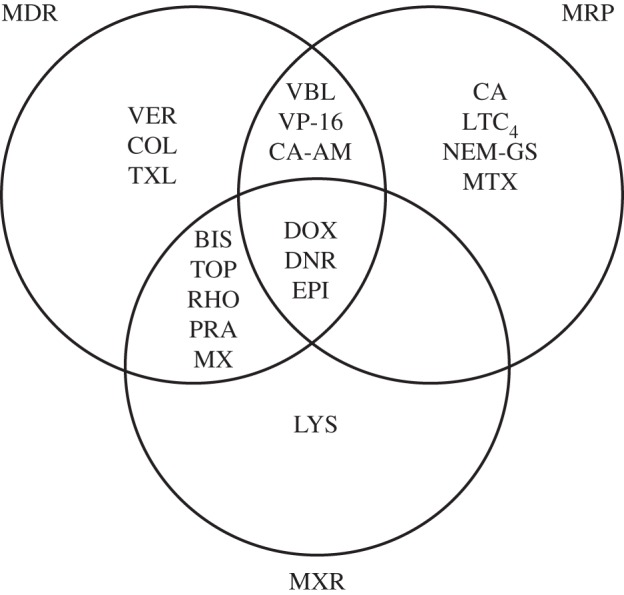
Substrate overlaps between the transporters P-glycoprotein/MDR1, multi-drug-resistance-associated protein (MRP) and mitoxantrone-resistance protein (MXR). The substrate and inhibitor profiles for the transporters were obtained from micrographs that showed the steady-state accumulation of fluorescent drugs (60 min incubation at 37°C); adapted from [7]. BIS, bisantrene; CA, calcein; CA-AM, calcein-AM ester; COL, colchicine; DNR, daunorubicin; DOX, doxorubicin; EPI, epirubicin; LTC_4_, leukotriene C4; LYS, LysoTracker; MTX, methotrexate; MX, mitoxantrone; NEM-GS, N-ethyl maleimide glutathione; PRA, prazosin; RHO, rhodamine 123; TXL, taxol; TOP, topotecan; VBL, vinblastine; VER, verapamil; VP-16, etoposide.

Decreased intracellular drug accumulation can result from a decrease in drug influx via drug solute carriers (SLC) [6] or from an increase in drug efflux via ATP-binding cassette (ABC) drug efflux pumps such as the P-glycoprotein (MDR1), multi-drug-resistance-associated protein (MRP) and mitoxantrone-resistance protein (MXR) [7]. These pumps are targeted by several anti-cancer drugs. The use of fluorescent calcein, which is an ABC transporter substrate, makes it possible to identify drugs that compete with calcein for the ABC transporter. Using similar methods, many chemotherapeutic drugs have been shown to be substrates/inhibitors of MDR1, MRP and MXR (figure 1). Some drugs used in chemotherapy have been specifically selected for use because they are not substrates for the ABC drug efflux pumps [8]. For example, this is true for the platinum drugs, which are used for treatment of solid tumours in more than 50% of all cancer patients. For these platinum-based drugs, drug resistance is often caused by decreased drug accumulation via the copper transporter 1 (CRT1), increased drug efflux via copper-transporting ATPases (ATP7A, ATP7B), increased detoxification of the drug by thiol-containing molecules within the cell [9–11] or, as described below, by evasion of programmed cell death (apoptosis).

In recent years, it has become increasingly clear that downregulation of ion channels and transporters is an important mechanism in the development of drug resistance via impairment of programmed cell death [12]. In this review, we discuss how drug resistance can develop through the modulation of membrane-bound ion transporters, limitation of cell shrinkage and, thus, impairment of apoptosis. We discuss the ion transporters that are involved in this process and try to clarify the mechanisms by which downregulation of channels can make the cancer cells apoptosis-resistant. It is noted that several reports have shown that ion channel overexpression can also be associated with apoptosis resistance. These examples will also be discussed below. Because modulation of ion channels is also involved in changes in cell migration and cell proliferation, we also briefly mention examples in which upregulation of ion channels contributes to tumour cell drug resistance. Finally, we point out that the cell-surface accessibility of ion channels suggests that they have strong potential as diagnostic and therapeutic targets in tumour treatment.

## 2. Evasion of drug-induced apoptosis

**Figure 2.**
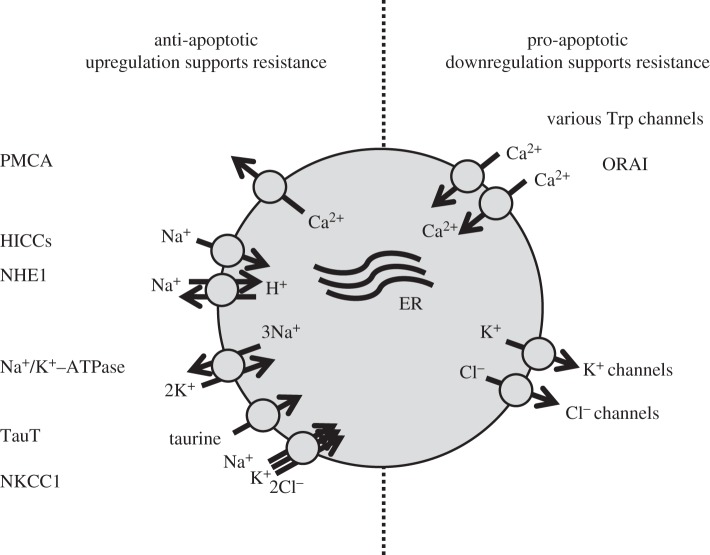
Anti- and pro-apoptotic plasma membrane-bound ion transporters involved in MDR. The anti-apoptotic transporters include the plasma membrane Ca^2+^ ATPase (PMCA), hypertonicity-induced cation channels (HICCs), the Na^+^/H^+^ exchanger (NHE1), the Na^+^/K^+^-ATPase, the Na^+^-dependent taurine transporter (TauT) and the 1Na^+^, 1K^+^, 2Cl^−^ cotransporter (NKCC1). The pro-apototic transporters include the membrane-bound Ca^2+^ channel (Orai1) and various transient receptor potential channels (Trps) and K^+^ and Cl^−^ channels.

A hallmark of apoptosis is cell shrinkage, which is also termed ‘apoptotic volume decrease’ (AVD); hence, disordered or altered cell volume regulation is associated with apoptosis (reviewed in [13]). AVD results from a loss of KCl via K^+^ and Cl^−^ channels and a concomitant loss of water [14–19], and it has turned out that downregulation of K^+^ channels [20] and Cl^−^ channels [19,21,22] courses resistance in cancer cells towards apoptosis. Cell shrinkage is usually followed by regulatory volume increase (RVI) [23,24] which counteracts AVD and thereby apoptosis [25,26]. The most important transport systems involved in RVI that have potential anti-apoptotic effects are the Na^+^, K^+^, 2Cl^−^ co-transporter NKCC1, the Na/K ATPase, cation channels and the Na^+^/H^+^ exchanger NHE1 [13,24] (figure 2, left-hand side). It has been demonstrated in several cell types that hypertonic cell shrinkage results in apoptosis (reviewed in [13]). For example, in NIH3T3 cells, caspase 3 activity increases fivefold following a twofold increase in extracellular osmolarity [27]. Bortner *et al*. [28] recently demonstrated that repetitive hypertonic exposure of lymphocytes resulted in a cell line with improved RVI and an attendant resistance towards shrinkage-induced apoptosis. In accordance with these observations, Chinese hamster ovary cells do not exhibit RVI because of lack of NHE1, and these cells are more prone to apoptosis compared with cells expressing NHE1 [25]. The activation of apoptosis following cell shrinkage may involve activation of p38/p53 signalling [27], CD95 death receptor trafficking to the plasma membrane [29], and inhibition of growth factor-mediated signalling [30]. The cooperation and coordination of signalling networks as a phenotypic hallmark of MDR is discussed in greater detail by Chen & Sikic [31].

## 3. Prevention of cell shrinkage protects against apoptosis

**Figure 3.**
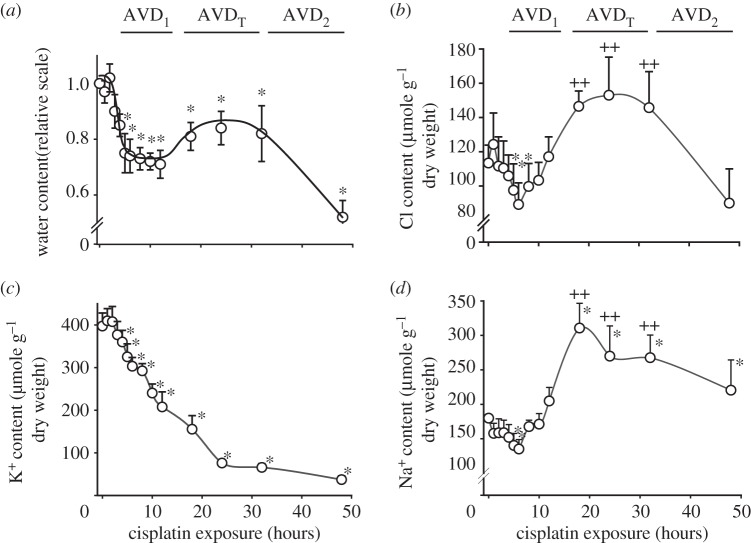
Time-dependent changes in cellular water content and ion content in Wt EATC following exposure to 5 µM cisplatin. (*a*) The water content (millilitre per gram cell dry weight) was normalized to values obtained prior to cisplatin exposure. (*b*) Cl^−^ content (micromole per gram cell dry weight) was obtained by Ag^+^ titration. (*c*,*d*) K^+^ and Na^+^ content was determined using emission flame photometry. The values are reported as means with the standard error of the mean. Asterisks (*) and plus symbols (++) indicate values that were significantly different from the initial control value. Adapted from [19].

**Figure 4.**
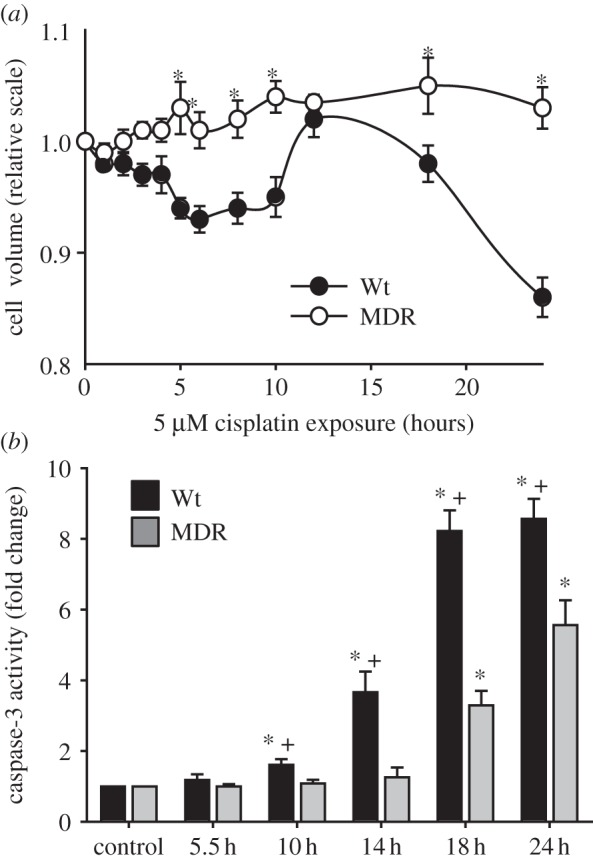
Changes in cell volume and caspase 3 activity in wild-type (Wt) and multi-drug resistant (MDR), EATC. (*a*) Cell volume was estimated by electronic cell sizing using the Coulter counter technique. (*b*) Caspase 3 activity was determined using a calorimetric assay to detect production of *p*-nitroanilide by cleavage of the substrate acetyl-Asp-Glu-Val-Asp *p*-nitroanilide. The values are reported as means with the standard error of the mean. In (*a*), asterisk (*) indicates a significant difference between Wt and MDR EATC cells. In (*b*), asterisk (*) indicates a significant difference compared with control, and plus symbol (+) indicates a significant difference between Wt and MDR EATC cells. Adapted from [19].

Experiments in Ehrlich ascites tumour cells (EATC) have demonstrated that the addition of a Ca^2+^ ionophore to cells in culture elicits a substantial net loss of KCl with concomitant cell shrinkage; this is followed by RVI, so that the cells regain their original volume within 10–15 min [32]. On the other hand, inducing a net loss of KCl in EATC with the chemotherapy drug cisplatin induces AVD, which has three stages. As seen in figure 3, these stages are designated AVD_1_, AVD_T_ and AVD_2_ and are characterized by a net loss of KCl, by a compensatory net uptake of NaCl and then by a net loss of KCl, respectively. AVD_T_ represents an unsuccessful RVI response in which the continuous loss of K^+^ reflects impaired function of the Na, K-ATPase [33]. Organic osmolytes are lost throughout the entire AVD process [19]. Figure 4*a* shows that multi-drug-resistant EATC (MDR EATC) obtained by treating EATC with daunorubicin for more than 70 passages [34] show no AVD_1_ response after the addition of cisplatin. While wild-type EATC (Wt EATC) enter apoptosis after addition of cisplatin, as reflected by a fourfold increase in caspase 3 activity within 14 h of the addition, MDR EATC show no significant increase in caspase 3 activity within the 14 h time frame (figure 4*b*). After 18 h of cisplatin exposure, both Wt and MDR EATC cells show eightfold and threefold increases in caspase 3 activities, respectively (figure 4*b*). Hence, the lack of AVD1 in MDR EATC correlates with prevention of apoptosis.

## 4. The role of ion channels in resistance to drug-induced apoptosis

Figure 2 (right-hand side) shows the pro-apoptotic ion channels. Notably, there is downregulation of these channels in MDR. These channels include the K^+^ and Cl^−^ channels, which are responsible for AVD, as well as Ca^2+^ channels, which are involved in Ca^2+^ influx and hence modulation of Ca^2+^-sensitive steps during apoptosis.

### (a) Cl^−^ channels

Reduction in volume-regulated anion current (VRAC) has been related to MDR in several cell lines [19,21,22,35]. However, VRAC activity is in HT-29 cells irrespective of MDR1 expression [36], and overexpression of MDR1 is accompanied by increases in VRAC current in the multi-drug-resistant cell line H69AR [37]. Gollapudi *et al*. [35] demonstrated that the Cl^−^ conductance was reduced in multi-drug-resistant HL60/AR cells compared with the HL60 parent cells, and that *in vitro* treatment of drug-sensitive HL60 cells with a Cl^−^ channel blocker resulted in increased resistance to daunorubicin. Likewise, Okada and co-workers [21] demonstrated that VRAC is absent in the multi-drug-resistant human epidermoid cancer cell line KCP-4 and that treatment with a histone deacetylase inhibitor causes partial restoration of VRAC activity and, concomitantly, cisplatin sensitivity. The effects in KCP-4 were blocked by simultaneous treatment of the cells with a VRAC channel blocker [21].

**Figure 5.**
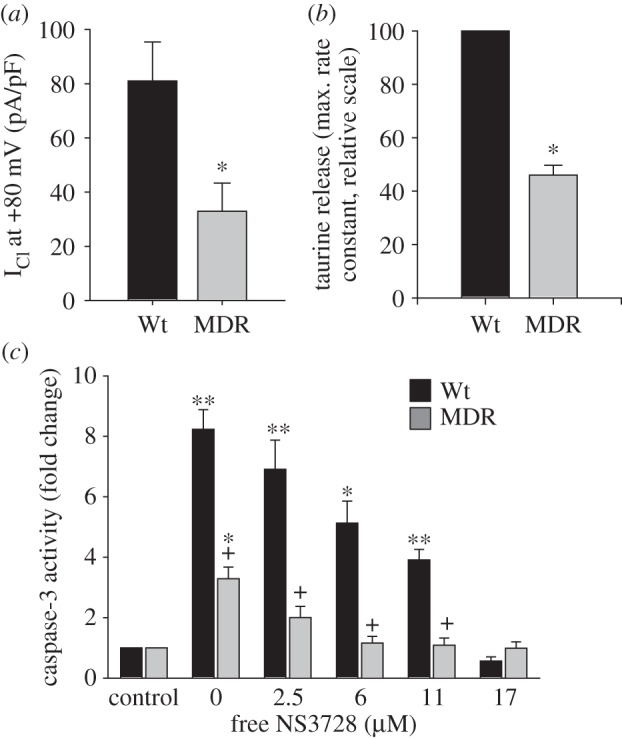
Downregulation of the volume-regulated Cl^−^ current/taurine release pathway in multi-drug resistant (MDR) Ehrlich ascites cells (EATC) and elimination of cisplatin-induced apoptosis following addition of the Cl^−^ channel blocker NS3728. (*a*) The volume-activated Cl^−^ current was measured using a whole-cell patch-clamp technique following hypotonic exposure (reduction of the extracellular medium to two-third of the isotonic value). (*b*) Volume-activated release of the organic osmolyte taurine was estimated as the maximal obtainable rate constant following hypotonic exposure. The MDR value is relative to the value in Wt cells. (*c*) Caspase 3 activity was measured using a calorimetric assay to detect production of *p*-nitroanilide by cleavage of the substrate acetyl-Asp-Glu-Val-Asp *p*-nitroanilide. NS3728 was added to block the Cl^−^ current, and the free concentration of NS3728 was determined using Centrifree YM-30 micropartition devices and ^14^C-labelled NS3728. In (*a*,*b*), asterisk (*) indicates significant differences compared with Wt EATC. In (*c*), asterisk (*) indicates a significant difference compared with control cells without cisplatin, and plus symbol (+) indicates a significant difference between Wt and MDR EATC cells. Adapted from [19].

As shown in figure 5*a,b*, VRAC, as well as the volume-sensitive leak pathway for organic osmolytes, is reduced in MDR EATC compared with Wt EATC. Addition of NS3728, which is an effective VRAC inhibitor [38], reduces the apoptotic response to cisplatin in a dose-dependent manner (figure 5*c*) in Wt and MDR EATC and at 17 µM NS3728 Wt EATC is as cisplatin resistant as the MDR EATC. This indicates that impaired VRAC activity in MDR EATC correlates with the impaired AVD response and with cisplatin resistance. Similarly, Min *et al*. [22] demonstrated that impaired VRAC activity contributes to cisplatin resistance in human lung adenocarcinoma (A549/CDDP) cells. Apoptosis is accompanied by DNA fragmentation and it has been shown that T cells lacking the type I transmembrane phosphatase CD45 have a reduced capacity to activate Cl^−^ channels and show less DNA fragmentation following induction of apoptosis via mitochondria perturbing agents [39]. It is suggested that loss of Cl^−^ increases DNA fragmentation. This is in agreement with the observation that inhibition of Cl^−^ channels blocks UV-C induced DNA degradation in human Jurkat cells [40]. However, data for the Jurkat cells indicate that the effect of Cl^−^ reduction is limited to intrinsic activation of apoptosis [40].

### (b) K^+^ channels

Potassium channel activity, and hence K^+^ loss, play an essential role in the initiation of apoptosis owing to (i) decay of the membrane potential and the associated Ca^2+^ influx; (ii) AVD; and (iii) activation of various enzymes involved in the apoptotic process [12,13,41,42]. Addition of clofilium, which is a TASK2 K^+^ channel blocker [24], prevents AVD and abrogates cisplatin-induced caspase 3 activity in Wt EATC [19]. Similarly, targeting the big conductance K^+^ channel with the inhibitor tetraethyl ammonium attenuates cisplatin-induced apoptosis in type I spiral ligament fibrocytes [43] and mouse neocortical neurons [44]. The TASK3 gene (Kcnk9) is overexpressed in several types of human carcinomas which has been associated with resistance towards apoptosis [45]. This is in contrast to what is seen in different glioma cell lines where application of the TASK3 channel opener isoflurane significantly reduces cell survival and the TASK channel blockers bupivacaine and spermine completely reverses this effect [46]. Downregulation of K^+^ channels as a resistance mechanism is observed in many malignant cancer cells—for example, the expression of Kv1.5 is suppressed in several cancer cell lines [47]. Furthermore, Han *et al*. [48] demonstrated that upregulation of Kv1.5 increases the K^+^ current and concomitantly the sensitivity to multiple chemotherapeutic drugs in gastric cancer cells (SGC7901), whereas downregulation of the channel enhances the drug-resistant phenotype. An additional example of downregulation of K^+^ channels in MDR cells is that the gene expression index for small (SK1/KCNN1) and intermediate (IK/KCNN4) conductance calcium-activated potassium channels is lower in MDR EATC (table 1). KCNN4 was recently associated with proliferation and invasion in colorectal cancer [49]. Moreover, the expression index for the K^+^ channel modulatory factor 1 (KCMF1) is reduced in MDR EATC (table 1). KCMF1 is broadly overexpressed in human cancer tissues, such as pancreatic carcinomas [50]. However, there are conflicting data as to whether KCMF1 has a pro-oncogenic [50,51] or a more tumour-suppressive [52] function, and its role in apoptosis needs to be investigated further.

**Table 1.** Downregulation in the expression of K^+^ channels in the MDR phenotype. The expression index was determined using the Affymetrix GeneChip Mouse Genome 430 2.0 microarray and the GeneChip Expression Analysis system. (T Litman & EK Hoffmann 2009, unpublished data.)

gene name	Wt EATC	MDR EATC
	gene expression index	
kcnn1	131 ± 2	108 ± 2
kcnn4	1451 ± 12	1246 ± 14
kcmf1	1450 ± 10	968 ± 15

### (c) Ca^2+^ channels

MDR can be achieved via downregulation of proteins involved in Ca^2+^ homeostasis, so targeting Ca^2+^ transporters in order to enhance the pro-apoptotic potential of malignant cells may be a useful strategy in the treatment of cancer. The calcium dependence of apoptosis is well described and seems to involve elevation of the intracellular Ca^2+^ concentration and decreases in the Ca^2+^ concentration in the endoplasmic reticulum (ER) for review [53,54]. To become resistant cancer cells could either reduce Ca^2+^ influx by downregulation of Ca^2+^ permeable channels and/or adapt to chronic-reduced ER Ca^2+^ [53]. The main plasma membrane-bound Ca^2+^ transporters that may be involved in the development of MDR include store-operated channels (SOC), transient receptor potential channels (Trps), voltage-gated Ca^2+^ channels and plasma membrane Ca^2+^ ATPases (PMCAs), which are briefly discussed below. The ER Ca^2+^-ATPase (Serca) and the inositol phosphate- (IP_3_-) sensitive receptor are not discussed in this review.

Induction of apoptosis in Bcl-2-overexpressing cells requires sustained Ca^2+^ influx via activated channels (SOCs), and downregulation of these channels seems to be a key component of the protective action of Bcl-2 against apoptosis in hormone-insensitive cancer cells [55]. Moreover, the apoptosis resistance of neuroendocrine (NE) differentiated prostate cancer cells seems to suggest that NE differentiation of prostate cancer epithelial cells involves reduction in the replenishment of the ER Ca^2+^ store, decreased expression of SERCA and substantial downregulation of SOCs [56]. SOCs are activated through a mechanism in which depletion of intracellular calcium stores leads to aggregation of STIM1, i.e. the Ca^2+^ sensor in ER, and Orai1, the membrane-bound Ca^2+^ channel protein. Reduced expression of Orai1, and, consequently, reduced SOC activity, prevents Ca^2+^ overload in response to pro-apoptotic stimuli and thus establishes the MDR phenotype in prostate cancer cells [57]. On the other hand, Faouzi *et al*. [58] suggest that Orai3 promotes apoptosis resistance in breast cancer cells. Several of the TRP channels have been discussed in relation to the regulation of Ca^2+^ influx during apoptosis and development of MDR, e.g. TRPC1, TRPV2 and TRPV6 [12,53]. The eventual role of the voltage-gated Ca^2+^ channels in MDR is complicated thus Ca_v_3.2 seems to be involved in apoptotic resistance in a prostate cancer cell line [12], whereas Cav3.1, which possess comparable biophysical properties to Cav3.2, promotes apoptosis in breast cancer cells [59].

## 5. Improvement of regulatory volume increase protects against apoptosis

Cell shrinkage is normally accompanied by an RVI response that reflects net uptake of Na^+^, K^+^ and Cl^−^ via the Na^+^/H^+^ exchanger, NKCC1, and via non-selective cation channels followed by exchange of cellular Na^+^ for extracellular K^+^ via the Na^+^/K^+^-ATPase [24]. As seen in figure 3, AVD_T_ represents an inadequate RVI response, i.e. the Na^+^/K^+^-ATPase is insufficient and the EATC cells continue to lose K^+^. The effect of inhibition of the Na^+^/K^+^-ATPase on apoptosis was reviewed previously [60]. Na^+^-dependent transporters for organic osmolytes contribute to the RVI response, while overexpression of the taurine transporter TauT protects kidney cells against cisplatin-induced apoptosis [61], TauT knockdown increases cisplatin-induced apoptosis in Ehrlich Lettré cells [62]. In agreement with this, Warskulat and co-workers demonstrated that mice lacking a functional TauT (TauT–/–) lack cellular taurine and become more prone to apoptosis, as seen in retinal degeneration [63,64].

### (a) Role of NKCC1, HICCs, NHE1 and PMCA

The literature concerning the role of NKCC1 and hypertonicity-induced cation channels (HICCs) in MDR is quite limited. In glioblastoma cancer cells, inhibition of NKCC1 with bumetanide augments temozolomid-induced AVD and apoptosis [65]. This raises the possibility that a combination of chemotherapeutic drugs with NKCC1 inhibitors might increase the efficiency of the chemotherapeutic treatment. In HeLa cells, HICCs rescue cells from staurosporine-elicited apoptosis [26].

In a number of cancer types, inhibition or knockdown of the Na^+^/H^+^ exchanger NHE1 has been shown to enhance chemotherapeutic cell death. In HeLa cells, which are a human cervical cancer-derived cell line, inhibition of RVI during hypertonic stress through application of NHE and anion exchanger blockers prolongs cell shrinkage and augments caspase-3 activation [25]. In agreement with this, hypertonic conditions induce apoptosis in NHE1-deficient PS120 fibroblasts, whereas transfection of HeLa cells with NHE1 restores RVI and prevents apoptosis [25]. In breast cancer cells, NHE1 is an essential player in paclitaxel-induced apoptosis; importantly, simultaneous inhibition of NHE1 results in synergistic potentiation of low-dose paclitaxel pro-apoptosis effects [66]. More recently, it was demonstrated that inhibition or knockdown of NHE1 sensitizes deltaNErbB2-expressing cells to cisplatin-induced apoptosis [67]. Overexpression of BCR-ABL and P-glycoprotein (Pgp) is a known mechanism underlying imatinib resistance, and NHE1 is an important target that has been implicated in the reversal of imatinib resistance in resistant leukaemia (K562) cell lines and in BCR-ABL-positive patient cells [68]. Notably, the role of NHE1 in drug resistance is not limited to its participation in RVI, since it is also involved in acidification of the extracellular nano-environment [69] and hence decreases the passive uptake of weakly basic chemotherapeutic drugs, e.g. doxorubicin, mitoxantrone, vincristine and vinblastine [70].

The plasma membrane Ca^2+^ ATPases (PMCAs) are low-capacity, high-affinity systems that export Ca^2+^ from the cytosol to the extracellular environment. There are four isoforms of PMCA: while PMCA1 and 4 are expressed ubiquitously, PMCA2 and 3 show more specific expression patterns [71]. Overexpression of PMCA seems to play a role in breast cancer progression by conferring resistance to apoptosis, and breast cancer patients with increased PMCA2 expression have a poor prognosis [72]. Baggott and co-workers [73] demonstrated that PMCA2-mediated inhibition of the calcineurin/NFAT signalling pathway is implicated in PMCA2-dependent apoptosis resistance in breast cancer cells.

## 6. Can upregulation of ion transport proteins that are important for proliferation contribute to multi-drug resistance?

Abnormal expression and/or activity of K^+^, Na^+^, Ca^2+^, Cl^−^ channels and TRP channels is involved in the growth and proliferation of cancer cells [74–76]. Thus, developing specific channel blockers represents a promising strategy for cancer treatment. It is not known whether upregulation of certain ion channels plays a role in MDR. TMEM16A (ANO1) is a Ca^2+^-activated Cl^−^ channel that is overexpressed in several carcinomas where it controls cell proliferation, migration and metastasis [76–79]. Targeting ANO1 has been proposed as a possible treatment for malignant tumours [80]. TMEM16F (ANO6) was also shown recently to be a Ca^2+^-activated Cl^−^ channel with delayed Ca^2+^ activation [81,82]; in addition, it has been associated with phospholipid scrambling and apoptosis [83]. Our group investigated whether ANO1 and ANO6 were upregulated during MDR development. Using QPCR with ARP as a reference gene, we found that ANO1 and ANO6 are strongly upregulated in MDR EATC compared with Wt EATC (ANO1 to ARP ratio: 0.00016 ± 0.00005 (*n* = 3) in Wt and 0.0021 (*n* = 2) in MDR; ANO6 to ARP ratio: 0.00002 ± 0.00001 (*n* = 3) in Wt and 0.0017 (*n* = 2) in MDR).

**Figure 6.**
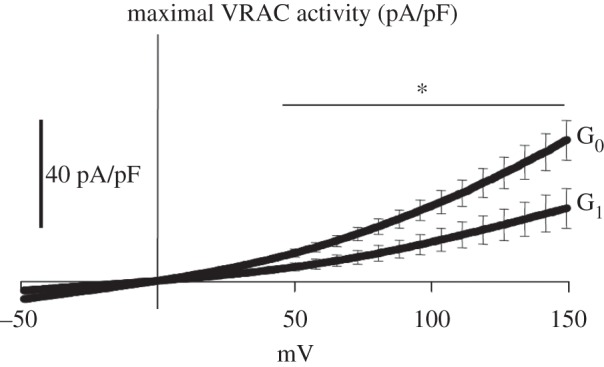
Cell cycle-dependent changes in maximal volume-regulated anion channel (VRAC) activity in ELA cells. The VRAC current was measured using a whole-cell patch-clamp technique as the Cl^−^ current in G_0_ and G_1_ phase ELA cells following exposure to hypotonic extracellular solution (190 mOsm) and at nominally zero [Ca^2+^]*_i_* (no added Ca^2+^, 10 mM EGTA in the pipette solution). The data shown are the I/V relationships based on the mean current density obtained from six to nine cells at each cell cycle phase; error bars indicate the standard error of the mean. Asterisk (*) indicates that the current densities in G_0_ are significantly different from those in G_1_ (*p* < 0.05). Adapted from [38].

It was demonstrated previously that the Cl^−^ channel CLC3 is upregulated in drug-resistant prostate cancer cells [84]. However, Cl^−^ channels are, as described above, generally considered to be pro-apoptotic and downregulated in MDR. These seemingly conflicting findings mandate further investigation. Indeed, cell proliferation and apoptosis both require activation of K^+^ and Cl^−^ channels. It is possible that activation of ion channels at localized/restricted areas of the plasma membrane (Ca^2+^-activated and agonist controlled) or sequentially activated channels (cell cycle dependent) are mainly involved in proliferation/migration, whereas a more global and persistent activation of channels (e.g. volume sensitive, voltage sensitive) is involved in apoptosis. Consistent with this hypothesis, we found that VRAC is downregulated in MDR EATC, which prevents apoptosis, whereas ANO1, which is normally associated with cell proliferation, is upregulated. VRAC is an example of an anion channel that has roles both in apoptosis and in cell cycle progression (VRAC activity decreases as cells go from G_0_ to G_1_) (figure 6). Hence, cells in G_0_ can either progress into apoptosis if VRAC levels increase (see §4) or continue into G_1_ and proliferate if VRAC levels decrease. In the latter case, reduced VRAC activity in MDR EATC (figure 5*a*) would both facilitate cell cycle progression and prevent apoptosis.

## 7. Conclusion

MDR is one of the most serious challenges when treating cancer using chemotherapy drugs. Many mechanisms are involved in MDR development, and the involvement of changes in the expression and function of ion channels and transport systems has only become clear in recent years. During the development of MDR, several pro-apoptotic ion channels are downregulated, while anti-apoptotic ion transporters are upregulated; these changes act to protect the cancer cells from cell death. However, there are also examples in which ion channels that are important for cell proliferation and migration are upregulated during the development of resistance. We do not yet have a clear picture of the differences between ion channels involved in apoptosis and ion channels involved in proliferation and migration. If ion channels are to be targeted by cancer therapies, then it is essential to know which channels are predominantly downregulated in MDR cells to prevent apoptosis and which predominantly promote growth and proliferation and thus are likely to be upregulated. One possibility is that the development of MDR involves sequential and localized upregulation of ion channels involved in proliferation and migration and a concomitant global and persistent downregulation of ion channels involved in apoptosis. To develop specific activators for the pro-apoptotic channels and specific blockers for the channels that are involved in tumour growth, migration and invasion, it is essential to distinguish between these types of channels and the mechanisms underlying their activation. Further research in this area is needed.

## Funding statement

This work was supported by The Danish Council for Independent Research (Natural Sciences and Medical Sciences), FP7 Curie Initial Training Network “IonTraC” (Ion Transport Proteins in Control of Cancer Cell Behaviour), and by the Novo Nordisk Foundation.
